# Porcine epidemic diarrhea virus induces Vero cell mitophagy by regulating ROS-mediated PINK1/Parkin signal pathway

**DOI:** 10.3389/fvets.2026.1892510

**Published:** 2026-07-20

**Authors:** Yuhe Jin, Ao Sun, Ye Jin, Xinxu Li, Mingyue Han, Haoran Zhang, Hanfan Qiao, Wenli Bai, Yue Zhang, Dahan Yang

**Affiliations:** 1 College of Animal Science and Technology, Inner Mongolia Minzu University, Tongliao, China; 2 Tongliao Animal Disease Prevention and Control Center, Tongliao, China; 3 State Key Laboratory for Animal Disease Control and Prevention, Lanzhou Veterinary Research Institute, Chinese Academy of Agricultural Science, Lanzhou, China; 4 Tongliao Academy of Agriculture and Animal Husbandry Sciences, Tongliao, China

**Keywords:** mitophagy, parkin, PINK1, porcine epidemic diarrhea virus, ROS

## Abstract

**Introduction:**

Porcine epidemic diarrhea (PED) is a highly contagious enteric disease caused by the porcine epidemic diarrhea virus (PEDV), with mortality rates exceeding 80% in piglets. While the pathogenesis of PEDV is well-documented, its relationship with mitophagy remains unclear.

**Methods:**

We used transmission electron microscopy (TEM) to observe ultrastructural changes in PEDV-infected Vero cells. Western blotting and biochemical assays were employed to analyze mitophagy-related markers (LC3-II, PINK1, Parkin, Beclin1) and oxidative stress indicators (ROS, MDA, antioxidant enzymes). The role of the PINK1/Parkin pathway was further validated through siRNA knockdown and overexpression.

**Results:**

PEDV infection was found to trigger PINK1/Parkin-dependent mitophagy, evidenced by the accumulation of damaged mitochondria and upregulation of mitophagy markers. This process was driven by ROS-induced oxidative stress, as ROS scavenging significantly attenuated mitophagy. Furthermore, we demonstrated that mitophagy facilitates PEDV replication, with Parkin expression acting as a critical regulator of this viral-induced autophagic pathway.

**Discussion:**

This study reveals that PEDV exploits PINK1/Parkin-mediated mitophagy via ROS accumulation to promote viral replication. These findings provide a scientific basis for understanding PEDV pathogenesis and offer a technical reference for optimizing high-titer virus production in vaccine development.

## 1 Introduction

Porcine epidemic diarrhea (PED) is an acute, highly contagious intestinal disease caused by the porcine epidemic diarrhea virus (PEDV) (1). The virus first appeared in the United Kingdom in the late 1970s (2). The primary clinical indications observed in infected herds encompass watery diarrhea, vomiting, dehydration, and, in severe cases, mortality (3). The disease exhibits a rapid transmission rate within herds; once pigs exhibit symptoms, the entire herd is rapidly affected. The incidence and mortality rates among piglets are significantly higher than those among adult pigs, resulting in major economic losses to the swine industry (4).

PEDV belongs to the genus *Alphacoronavirus* within the family *Coronaviridae* and is an enveloped, single-stranded positive-sense RNA virus (5). PEDV is an enterotropic virus that enters the animal’s body via the digestive tract. It primarily affects the jejunum and ileum, damaging the intestinal epithelial cells (IECs) of pigs (6, 7). *In vitro* studies have demonstrated the capacity of PEDV to effectively infect two distinct cell lines: porcine intestinal epithelial cells (IPEC-J2) and African green monkey kidney cells (Vero) (8–11). Previous studies have shown that PEDV infection can trigger oxidative stress and apoptosis in Vero cells (12), and induce endoplasmic reticulum stress and autophagy through PERK and IRE1 pathways in IECs and Vero cells, with autophagy promoting viral replication (13). In this study, we chose Vero cells as the initial model because they are highly sensitive to PEDV and have been widely used in virus isolation and basic mechanism research (14, 15). Although research into the intracellular replication mechanisms of PEDV is ongoing and new findings continue to emerge, its pathogenic mechanisms and the role of autophagy in this process remain unclear.

Mitochondria are key organelles for cellular oxidation and energy metabolism (16, 17). Viral infections can induce reactive oxygen species (ROS), leading to mitochondrial oxidative damage and dysfunction (18). Accumulating evidence indicates that ROS generated during infection can trigger mitochondrial dysfunction and activate mitophagy, which remodels the mitochondrial network to limit damage and reshape cellular redox status—processes that may influence viral replication (19, 20). Within cells, ROS are byproducts of electron transfer, primarily originating from the substrate side of the mitochondrial inner membrane respiratory chain (21). Excessive ROS production promotes mitochondrial permeability transition, disrupts electron transport, reduces ATP production, and further amplifies ROS generation, resulting in a feed-forward decline in cellular homeostasis. Therefore, ROS–mitophagy signaling has the potential to affect the intracellular environment required for viral gene expression, organelle homeostasis, and replication.

Excessive production and accumulation of reactive ROS can promote the oxidation of reduced coenzyme I (NADH), coenzyme II (NADPH), and glutathione (GSH) bound to mitochondrial double membrane porins, leading to the opening of these porins, decoupling of the mitochondrial electron transport chain, reduced ATP production, and rupture of the outer mitochondrial membrane; in turn, damaged mitochondria generate more ROS, further exacerbating the decline in. In the present study, transmission electron microscopy and fluorescence microscopy were utilized to examine the morphology, ultrastructure, ROS levels, and mitochondrial membrane potential (MMP, ΔΨm) of Vero cells infected with PEDV, which ultimately promotes the activation of mitophagy (22). Mitophagy, a specialized form of cellular autophagy, plays a crucial role in protecting against oxidative damage and maintaining cellular energy metabolism (23, 24). Among several mitophagy routes, the PINK1/Parkin pathway is the best characterized ubiquitin-dependent mechanism and is associated with loss of mitochondrial membrane potential (MMP) (25–27). In healthy mitochondria, PINK1 is transported into the mitochondria via the outer membrane translocase (TOM) and inner membrane translocase (TIM17/TIM23) precursor pathways, and is then rapidly degraded at the inner mitochondrial membrane (IMM) (28). Under oxidative stress conditions, excessive accumulation of ROS leads to a decrease in MMP, which impedes protein import and reduces TIMM23 levels. PINK1 aggregates at the damaged outer mitochondrial membrane and recruits cytoplasmic Parkin to the damaged mitochondria. This process subsequently phosphorylates Parkin and activates Parkin’s E3 ligase via ubiquitination, thereby mediating the degradation of various outer mitochondrial proteins, such as VDAC1, Mitofusin 1 (Mfn1), and Mitofusin 2 (Mfn2), among other mitochondrial outer membrane proteins. This process subsequently recruits the SQSTM/p62 adaptor protein to bind to the autophagy marker ATG8/LC3 family proteins via the LC3-interacting region (LIR), promoting the formation of mitophagosomes and initiating mitophagy (29, 30). This mechanism plays a key role in mitophagy in mammals (31).

There is mounting evidence that autophagy plays a pivotal role in both promoting and inhibiting viral activity (32, 33). For example, classical swine fever virus (CSFV) induces ROS production that triggers PINK1/Parkin-mediated mitophagy and promotes viral replication (34), while porcine circovirus 2 (PCV2) induces PINK1/Parkin-dependent mitophagy through Drp1 phosphorylation (35). Studies have shown that during PEDV infection, the virus induces mitophagy; however, the specific regulatory mechanisms and its exact role in viral replication remain unclear (36). Therefore, the objective of this study is to investigate the role of mitophagy in PEDV infection and to elucidate the regulatory mechanisms of mitophagy, ROS, and the PINK1/Parkin signaling pathway during viral infection of host cells. This study will provide new insights for identifying drug targets for the prevention and treatment of PEDV.

## 2 Materials and methods

### 2.1 Reagents

The chemical reagents employed in this study were: mitochondria-targeted ROS scavenger (MitoQ); carbonyl cyanide m-chlorophenyl hydrazine (CCCP); and cyclosporine A (CsA). These reagents were dissolved in dimethyl sulfoxide and stored at −80 °C. The primary antibodies used in this study are as follows: rabbit polyclonal an-ti-PINK1 (Abcam, ab216144); rabbit polyclonal anti-Parkin (Abcam, ab77924); rabbit polyclonal anti-TIMM23 (Abcam, ab230253); rabbit polyclonal anti-p62/SQSTM1 (Abcam, ab109012); rabbit polyclonal anti-LC3B (Abcam, ab192890); rabbit polyclonal anti-Beclin-1 (Abcam, ab207612); and rabbit polyclonal anti-GAPDH (Abcam, ab181602). Rabbit polyclonal anti-PEDV-N is an antibody stored in our laboratory. The secondary antibody for Western blotting was horseradish peroxidase-labeled goat anti-rabbit antibody (Abcam, ab6721).

### 2.2 Cell culture

The Vero E6 cells utilized in the experiment were cultivated in our laboratory. Vero E6 cells (African green monkey kidney cell line) were cultured in high-glucose ulbecco’s Modified Eagle’s Medium (DMEM) supplemented with 10% fetal bovine serum (FBS). All cells were cultivated in an incubator maintained at 37 °C and 5% CO₂. The Vero E6 cells utilized in this study were authenticated by Short Tandem Repeat (STR) profiling analysis to ensure cell identity and the absence of cross-contamination. Cells were maintained at low passage numbers (below passage 25) during the experimental procedures. And, all cell lines used in this study were routinely screened and confirmed to be negative for mycoplasma contamination using a PCR-based mycoplasma detection kit.

### 2.3 Virus infection

The PEDV HLJ2021 GIIc strain utilized in this study was provided by Prof. Guangxing Li of Northeast Agricultural University (37). Vero E6 cells, cultured *in vitro* until they reached a stable state, were divided into a blank control group and a PEDV-infected group. The original culture medium was discarded, and the cells were washed with phosphate-buffered saline (PBS). DMEM cell culture medium was added to the blank control group, while PEDV virus solution with an MOI of 0.1 was added to the PEDV-infected group. After 1 h of incubation, the viral culture medium was discarded, and the cells were washed with PBS, followed by the addition of complete culture medium (high-glucose DMEM supplemented with 2% FBS). All cells were placed in an incubator set to 37 °C and with 5% CO₂ for 6, 12, 18, and 24 h.

### 2.4 Biochemical intervention

Vero E6 cells were inoculated and cultured until 80–90% of them adhered to the surface. The cells were divided into several independent groups. In the study on the effect of ROS on PEDV replication, four groups were set up, namely, the PEDV infection group treated only with PEDV (MOI = 0.1), the MitoQ intervention group treated with 1 μM targeted mitochondrial reactive oxygen species scavenger MitoQ, the MitoQ intervention control group without PEDV treatment but using 1 μM targeted mitochondrial reactive oxygen species scavenger MitoQ, and the blank control group. In the study on the effect of mitochondrial autophagy on PEDV replication, six groups were set up, namely the PEDV infection group treated only with PEDV, the CCCP group treated with 10 μM mitochondrial autophagy inducer CCCP, the CCCP intervention control group without PEDV treatment but using 10 μM mitochondrial autophagy inducer CCCP, the CsA group treated with 5 μM mitochondrial autophagy inhibitor CsA, the CsA intervention control group without PEDV treatment but using 5 μM mitochondrial autophagy inhibitor CsA, and the blank control group. The infection multiplicity of the groups treated with PEDV was all 0.1, and the intervention control groups did not add the virus solution, but were treated with the same volume of DMSO, while the blank control groups were only treated with DMEM. After the treatment, all cells were cultured in a 37 °C, 5% CO₂ incubator for 6, 12, 18, and 24 h, and then collected.

### 2.5 Transmission electron microscopy (TEM)

Collect Vero E6 cells that have been cultured for 24 h from the blank control group and the PEDV-infected group, respectively. Upon collection, wash the cells three times with pre-chilled PBS to remove residual culture medium. Then, gently scrape the cells from the bottom of the culture dishes using a cell scraper, and transfer them to clearly labeled 1.5 mL EP tubes. Place the EP tubes in a benchtop refrigerated centrifuge and centrifuge at 1,000 rpm/min for 5 min. Remove the supernatant, add an appropriate amount of pre-chilled, 2.5% glutaraldehyde fixative to the cell pellet, mix, and incubate in a 4 °C refrigerator in the dark for 3 h. After fixation, examine the samples using a transmission electron microscope.

### 2.6 Immunofluorescence microscopy

Intracellular mitochondrial ROS production was measured using a reactive oxygen species assay kit (Beyotime, S0033S) in conjunction with the fluorescent probe 6-carboxy-2′,7′-dihydrofluorescein diacetate (DCFH-DA). Cells were placed in 10 μM DCFH-DA and cultured in serum-free medium at 37 °C for 30 min. The cells were then washed three times with pre-cooled PBS, observed under a fluorescence microscope, and images were captured. Five random fields were selected for quantitative analysis. Similarly, MMP was measured using the MMP Assay Kit (Beyotime, C2006). PEDV-infected cells were incubated with JC-1 probe in serum-free medium at 37 °C for 20 min. After three washes with PBS, the cells were observed under a fluorescence microscope to assess the fluorescence of JC-1 aggregates or monomers, images were captured, and quantitative analysis was performed.

### 2.7 Oxidation and antioxidant level testing

After 24 h of infection or drug treatment, Vero cells were washed three times with cold PBS, harvested by scraping, and lysed on ice using the lysis buffer provided in the respective kit. The cell suspension was then homogenized via ultrasonication and centrifuged at 12,000 × g for 10 min at 4 °C to collect the cell lysates (supernatants of lysed cells). The assessment of cellular oxidation and antioxidant markers, including malondialdehyde (MDA), superoxide dismutase (SOD), glutathione peroxidase (GPX), and total antioxidant capacity (T-AOC), was conducted in accordance with the protocols outlined in the accompanying kits. The Lipid Oxidation Assay Kit (Beyotime, S0131S; sensitivity: 0.1 nmol/mL); the Total SOD Activity Assay Kit (Beyotime, S0101S; detection limit: 0.5 U/mL); the Total Antioxidant Capacity Assay Kit (Solarbio, BC1310; sensitivity: 0.05 nmol/mL); and the Glutathione Peroxidase Activity Assay Kit (Solarbio, BC1190; detection limit: 5 mU/mL).

### 2.8 Overexpression of the Parkin gene and siRNA–mediated silencing in Vero E6 cells

Parkin gene silencing and overexpression plasmids were constructed by Wuhan MiaoLingBio (MiaoLingBio, China). Vero E6 cells were seeded in 6-well plates and cultured until 70% confluence, at which point they were transfected with the constructed Parkin gene silencing and overexpression plasmids. The following groups were established: To determine the regulatory role of Parkin, cells were assigned to six groups: the blank control group: cells treated with DMEM cell culture medium (without virus infection or any other intervention); PEDV group: cells infected with PEDV viral solution at an MOI of 0.1; Vector group: cells transfected with an empty plasmid vector and infected with PEDV (MOI = 0.1); Parkin group: cells transfected with a Parkin gene overexpression plasmid and infected with PEDV (MOI = 0.1). sh-NC group: cells transfected with a non-targeting control short hairpin RNA (shRNA) and infected with PEDV (MOI = 0.1). sh-Parkin group: cells transfected with a Parkin gene-specific shRNA (silencing plasmid) and infected with PEDV (MOI = 0.1). Cells from all groups were then cultured for an additional 24 h in a 37 °C, 5% CO₂ incubator before being harvested.

### 2.9 Western blotting

The cells were washed with PBS, then protease inhibitor PMSF and protein lysis buffer were added, and the cells were lysed on ice for 30 min. After centrifugation at 4 °C and 12,000 rpm for 10 min, the supernatant was collected as whole-cell lysate (WCL). Subsequently, the protein concentration of the WCL was determined. The protein sample was separated using 12.5% sodium dodecyl sulfate–PAGE and transferred to a PVDF membrane. The PVDF membrane was soaked in 5% skim milk at room temperature for 2 h, then incubated with the specific primary antibody overnight at 4 °C, followed by incubation with the specific secondary antibody for 1 h at room temperature. After washing, the Western blot (WB) results were visualized using ECL rea-gents and imaged on a chemiluminescence imaging system. ImageJ 1.5.0 software was used to analyze the grayscale values of the images, and the relative expression levels of the target proteins were calculated using GAPDH as an internal control.

### 2.10 Statistical analysis

**Figure 1:**
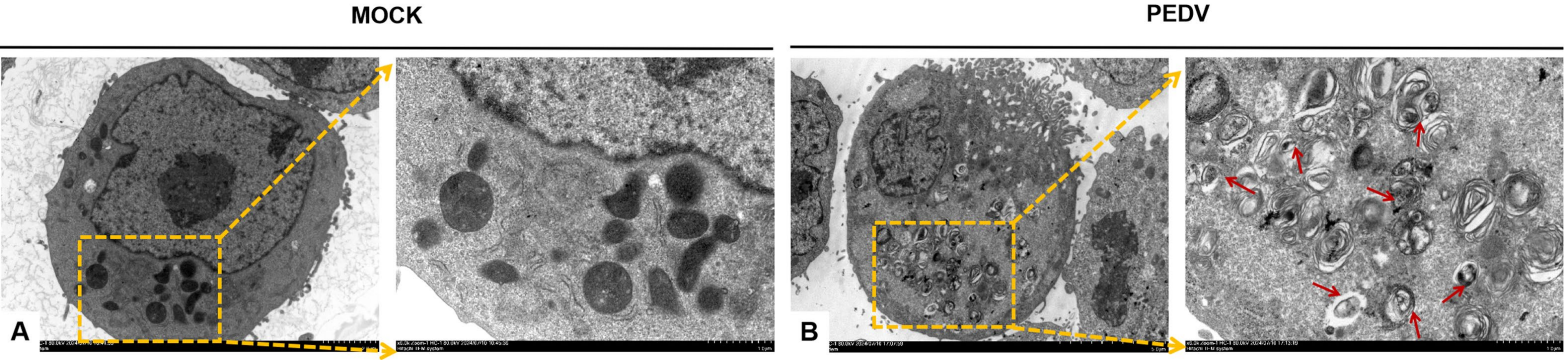
Infection with porcine epidemic diarrhea virus (PEDV) induces the formation of mitophagosomes. **(A)** Transmission electron microscopy (TEM) revealed normal mitochondrial structures in Vero cells 24 h after culture. Bar = 2.0 μm (left panel); Bar = 1.0 μm (right panel). **(B)** TEM showing Vero cells after 24 h of culture with PEDV (MOI = 0.1) mitochondrial swelling (red arrows) and loss of cristae structure (red arrows). Bar = 2.0 μm (left panel); Bar = 1.0 μm (right panel).

All data are expressed as mean ± standard deviation (SD). To compare differences between groups, the following statistical methods were employed in this study: For comparisons between the control group and the infected group at multiple time points (e.g., 6, 12, 18, and 24 h), two-way ANOVA was used, followed by Sidak’s multiple comparison test (e.g., to analyze protein expression levels over time in Figure 1); For comparisons of data among multiple treatment groups (e.g., different biochemical intervention groups or genetic manipulation groups) at a single time point (e.g., 24 h post-infection), one-way ANOVA was used, followed by Tukey’s *post hoc* test. All statistical analyses were performed using GraphPad Prism 10 software. Significance levels were set as follows: *p < 0.05, **p < 0.01, ***p < 0.001, ****p < 0.0001; ns indicates *p* ≥ 0.05.

**Figure 2:**
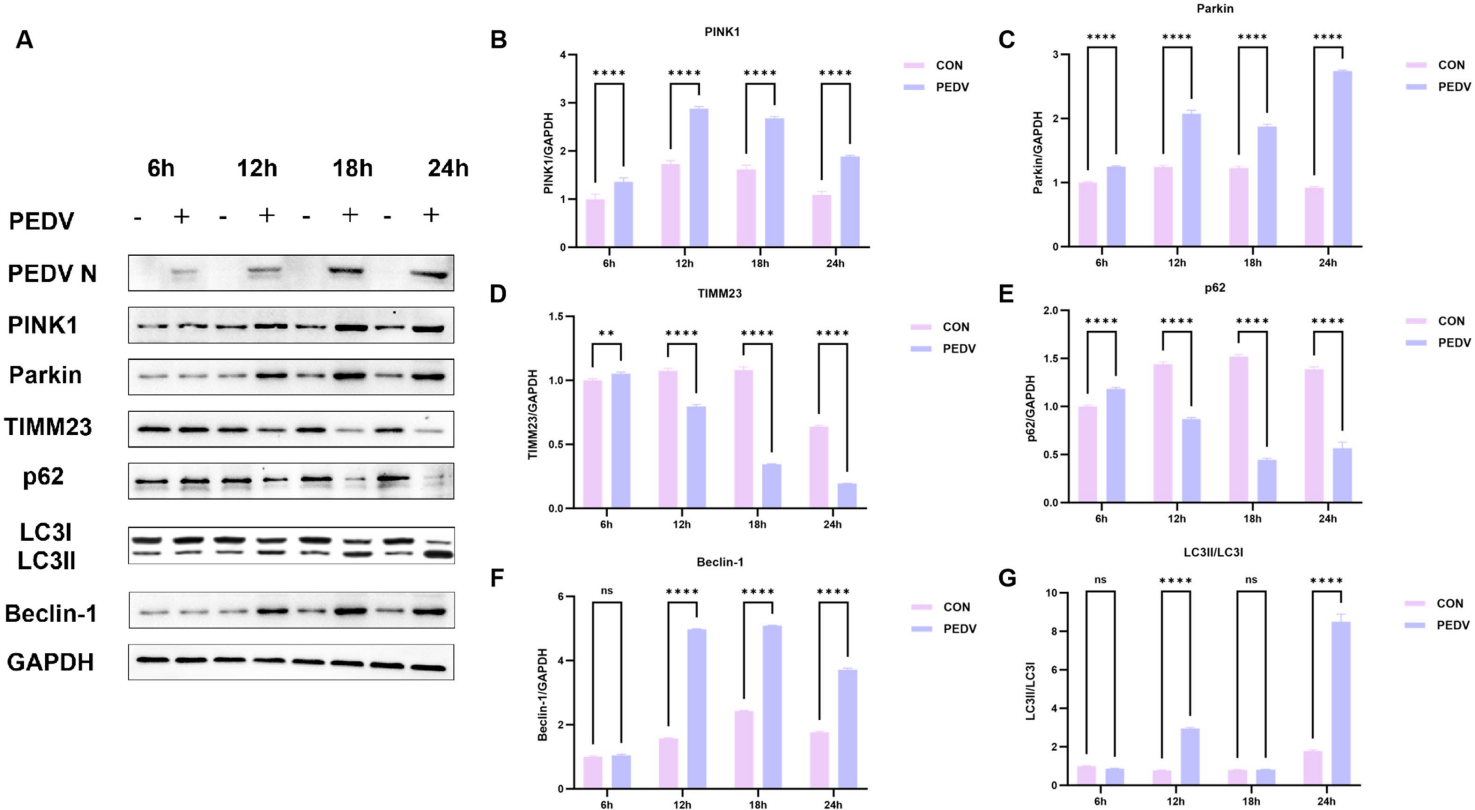
The Effects of PEDV Infection on the PINK1/Parkin Pathway. **(A)** Representative Western blot images showing PINK1, Parkin, TIMM23, p62, Beclin-1, LC3II/I protein levels. **(B–G)** The relative expression levels of PINK1, Parkin, TIMM23, p62, LC3II/I, and Beclin-1 in each group of Vero cells. Grayscale values of the protein levels normalized to GAPDH. Each biological replicate was assayed in technical triplicate. Data are presented as mean ± SD. Comparisons between two groups were performed using a two-way ANOVA analysis. (**p < 0.01, ****p < 0.0001; ns, not significant).

## 3 Results

### 3.1 PEDV infection induces mitophagy in cells

We examined the mitochondrial ultrastructure and autophagosome formation in Vero E6 cells at 24 h post-PEDV infection (MOI = 0.1) using TEM to assess the occurrence of mitophagy. TEM analysis revealed that tubular mitochondria with well-defined cristae were observed in cells from the MOCK group after 24 h of culture (Figure 2A). In contrast, at 24 h post-infection (hpi), PEDV-infected cells exhibited marked mitochondrial swelling and disruption of cristae structures (Figure 2B). Furthermore, we confirmed a significant increase in the accumulation of damaged, abnormal mitochondria and double-membrane mitophagosomes in PEDV-infected cells at 24 hpi compared to the mock control. These findings suggest that PEDV causes mitochondrial structural damage and induces mitophagy in Vero E6 cells.

### 3.2 PEDV infection induces a PINK1/Parkin-dependent autophagy pathway

In order to study the pathways through which PEDV infection induces autophagy, we employed the Western blot analysis method to detect the expression levels of autophagy-related proteins at 6, 12, 18, and 24 h after PEDV infection (Figure 1A). The results confirmed that PEDV infection upregulated the protein expression levels of PINK1 and Parkin in Vero cells (*p* < 0.05; Figures 1B,C). At 12 h post-infection, the expression levels of the mitochondrial inner membrane transporter TIMM23 and the adaptor protein p62 were both significantly reduced (Figures 1D,E), while the expression level of Beclin-1 was significantly increased 12hpi (*p* < 0.05; Figure 1F). In addition, the ratio of LC3 II to LC3 I in Vero cells was significantly increased at 12 and 24hpi (*p* < 0.05; Figure 1G). Taken together, these results indicate that PEDV-induced mitophagy is mediated by the PINK1/Parkin pathway and proceeds via a complete autophagic flux.

### 3.3 PEDV infection causes oxidative damage to cells

**Figure 3:**
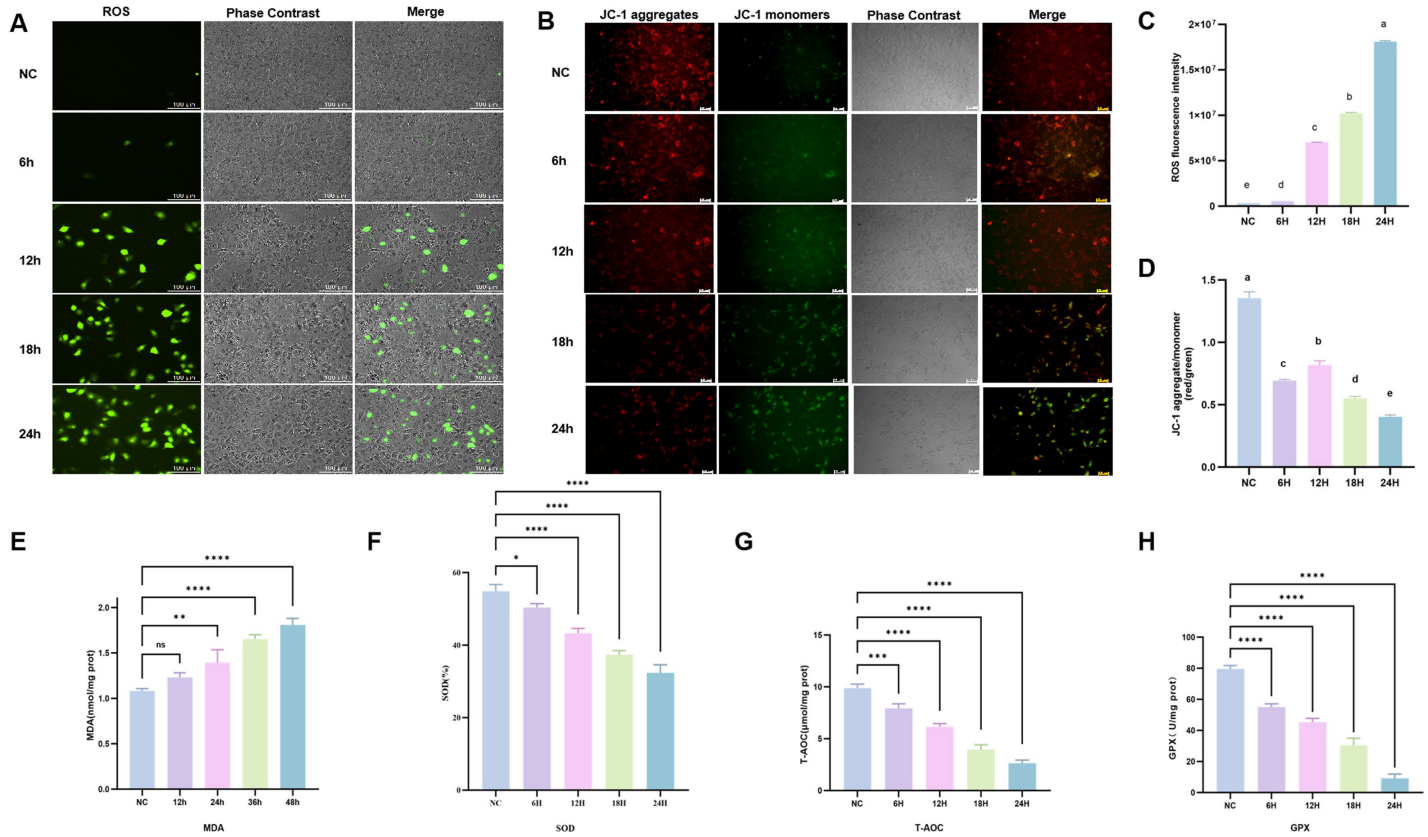
The Effects of PEDV Infection on Cellular Oxidation and Mitochondrial Membrane Potential(MMP). **(A)** Immunofluorescence microscopy images show the ROS accumulation in VERO cells from the blank control group and the PEDV-infected group. The green fluorescence signal represents the intracellular ROS levels detected by DCFH-DA. Bar = 100 μm. **(B)** Immunofluorescence microscopy images show the expression of JC-1 in Vero cells from the blank control group and the infected group. Green fluorescence represents JC-1 monomers, while red fluorescence represents JC-1 aggregates. Bar = 0.01 mm. **(C)** Relative fluorescence intensity of ROS. **(D)** Relative fluorescence intensity of JC-1 staining. **(E–H)** Compared with the blank control group, the expression levels of malondialdehyde (MDA), glutathione peroxidase (GPX), total antioxidant capacity (T-AOC), and the activity of superoxide dismutase (SOD) were measured in PEDV-infected Vero cells at different time points. Data are presented as mean ± SD. Statistical significance was obtained by one-way ANOVA. (*p < 0.05, **p < 0.01, ***p < 0.001, ****p < 0.0001; ns, not significant). More groups with different letters represented statistically significant differences, p < 0.05; the same letter indicated that there was no significant difference between different groups, *p* > 0.05.

To investigate the effects of PEDV infection on host cell mitochondria, we measured intracellular mitochondrial ROS production using DCFH-DA and assessed MMP using JC-1 dye. The results showed that 6, 12, 18, and 24 h after PEDV infection, intracellular ROS levels were significantly elevated (Figures 3A,C), and MMP was significantly reduced (Figures 3B,D). In addition, we used commercial kits to measure the levels of MDA, SOD, GPX, and T-AOC in the cells. The results showed that intracellular MDA levels were significantly elevated (*p* < 0.05; Figure 3E), while SOD, GPX, and T-AOC levels were significantly reduced (*p* < 0.05; Figures 3F–H). This indicates that PEDV infection increases intracellular ROS levels, leading to a decrease in MMP, accumulation of lipid peroxides, and oxidative damage to cells, which in turn causes mitochondrial damage. This may be the mechanism by which PEDV infection induces mitophagy.

### 3.4 The regulatory role of ROS in PEDV-induced mitophagy and viral replication

**Figure 4:**
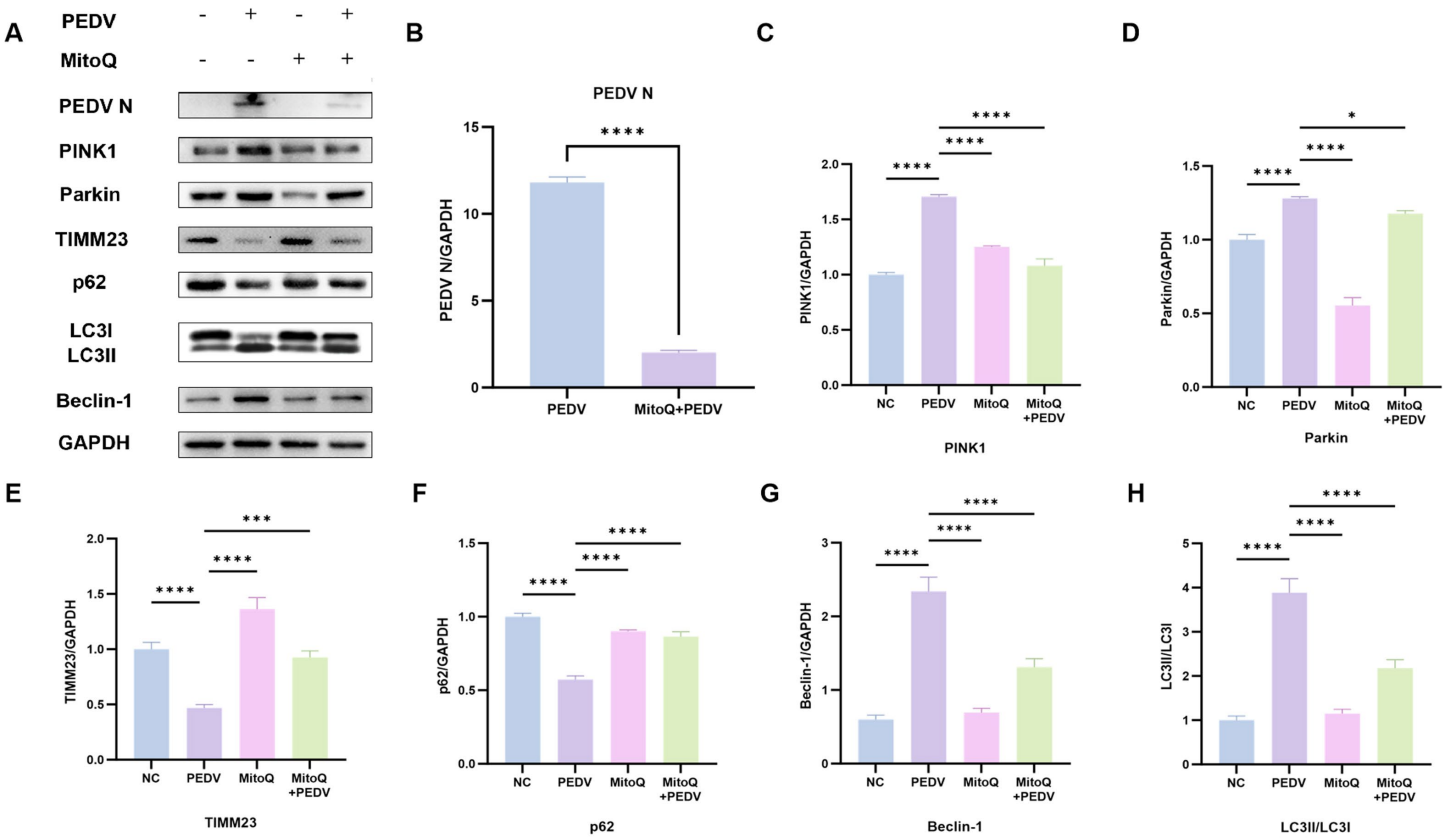
The Regulatory Role of ROS in PEDV-Induced Mitophagy and Viral Replication **(A)** Representative Western blot images showing PEDV N, PINK1, Parkin, TIMM23, p62, LC3II/I, Beclin-1 protein levels in Vero cells of each group. **(B)** Compared with the PEDV-infected group, the relative protein expression level of PEDV N in the Vero cells of the mitochondria-targeted ROS scavenger (MitoQ) group. **(C–H)** The relative expression levels of PINK1, Parkin, TIMM23, p62, LC3II/I, and Beclin-1 in each group of Vero cells. Grayscale values of the protein levels normalized to GAPDH. Each biological replicate was assayed in technical triplicate. Data are presented as mean ± SD. Compared with the control group, were performed using a one-way ANOVA analysis. (*p < 0.05, ***p < 0.001, ****p < 0.0001).

The objective of this study was to ascertain the effect of ROS on PEDV replication. To this end, we selected Vero cells infected with PEDV for 24 h and pretreated them with the MitoQ, followed by Western blot analysis to detect the expression levels of relevant proteins (Figure 4A). The results of the western blot analysis demonstrated a significant reduction in the expression of the PEDV N protein in Vero cells that had been pretreated with MitoQ (*p* < 0.05; Figure 4B). In addition, the suppression of ROS led to a decrease in the expression of PINK1 (*p* < 0.05; Figure 4C) and Parkin (*p* < 0.05; Figure 4D) in Vero cells. In comparison with the PEDV control group, the expression levels of the inner mitochondrial membrane transporter TIMM23 (*p* < 0.05; Figure 4E) and the adaptor protein p62 (*p* < 0.05; Figure 4F) were significantly elevated in MitoQ-treated cells, while the LC3II/LC3I ratio (*p* < 0.05; Figure 4G) and Beclin-1 (*p* < 0.05; Figure 4H) expression levels were significantly reduced. Consequently, the findings of this study suggest that the suppression of ROS can effectively hinder PEDV replication.

### 3.5 The influence of mitophagy on the replication of PEDV

**Figure 5:**
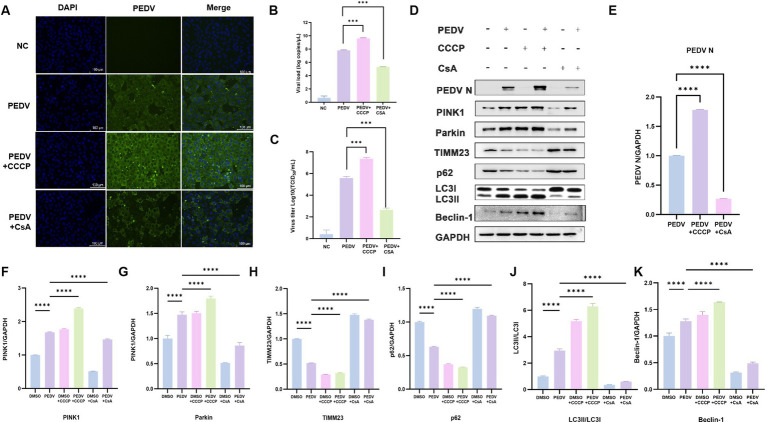
The effect of mitophagy on PEDV replication. **(A)** Immunofluorescence microscopy images showing the expression levels of PEDV-N in Vero cells from each group. The green fluorescent signal represents PEDV-N; the blue fluorescent signal represents the cell nuclei. Bar = 100 μm. **(B)** Viral load in Vero cells in the carbonyl cyanide m-chlorophenyl hydrazine (CCCP) and cyclosporine A (CsA) groups, compared to the PEDV-infected group. **(C)** Viral titers in Vero cells in the CCCP and the CsA groups, compared to the PEDV-infected group. **(D)** Representative Western blot images showing PEDV N, PINK1, Parkin, TIMM23, p62, LC3II/I, Be-clin-1 protein levels in Vero cells of each group. **(E)** Compared with the PEDV-infected group, the relative protein expression level of PEDV N in the Vero cells of the CCCP and CsA groups. **(F–K)** The relative expression levels of PINK1, Parkin, TIMM23, p62, LC3II/I, and Beclin-1 in each group of Vero cells. Grayscale values of the protein levels normalized to GAPDH. Each biological replicate was assayed in technical triplicate. Data are presented as mean ± SD. Compared with the control group, were performed using a one-way ANOVA analysis. (***p < 0.001, ****p < 0.0001).

This study aimed to determine the effect of mitophagy on PEDV replication. To achieve this, we selected Vero cells infected with PEDV for 24 h and pretreated them with a mitophagy inducer (CCCP) and a mitophagy inhibitor (CsA), and observations were made using immunofluorescence microscopy (Figure 5A), then used Western blotting to detect the expression levels of PEDV N proteins. Immunofluorescence microscopy revealed that, compared to the PEDV group, viral load and viral titer were significantly elevated in the CCCP group, while they were significantly reduced in the CsA group (*p* < 0.05; Figures 5B,C). Concurrently, Western blot results showed (*p* < 0.05; Figure 5D) that the expression of the PEDV N protein was markedly increased in Vero cells pretreated with CCCP; in contrast, the expression of the PEDV N protein was significantly reduced in cells treated with CsA (*p* < 0.05; Figure 5E). Furthermore, mitophagy promoted the expression of PINK1 and Parkin in Vero cells (*p* < 0.05; Figures 5F,G). Compared with the PEDV control group, the expression levels of the mitochondrial inner membrane transporter TIMM23 and the adaptor protein p62 were significantly reduced in CCCP-treated cells (*p* < 0.05; Figures 5H,I), while the LC3II/LC3I ratio and the expression level of Beclin-1 were significantly increased (*p* < 0.05; Figures 5J,K). In contrast, in CsA-treated cells, the expression levels of PINK1 and Parkin were significantly reduced, while TIMM23 and p62 were significantly elevated, and the LC3II/LC3I ratio and Beclin-1 protein expression levels were significantly decreased. Therefore, our results indicate that mitophagy increases PEDV viral titers and promotes PEDV replication.

### 3.6 The regulatory role of the PINK1/Parkin pathway in PEDV-induced mitophagy

**Figure 6:**
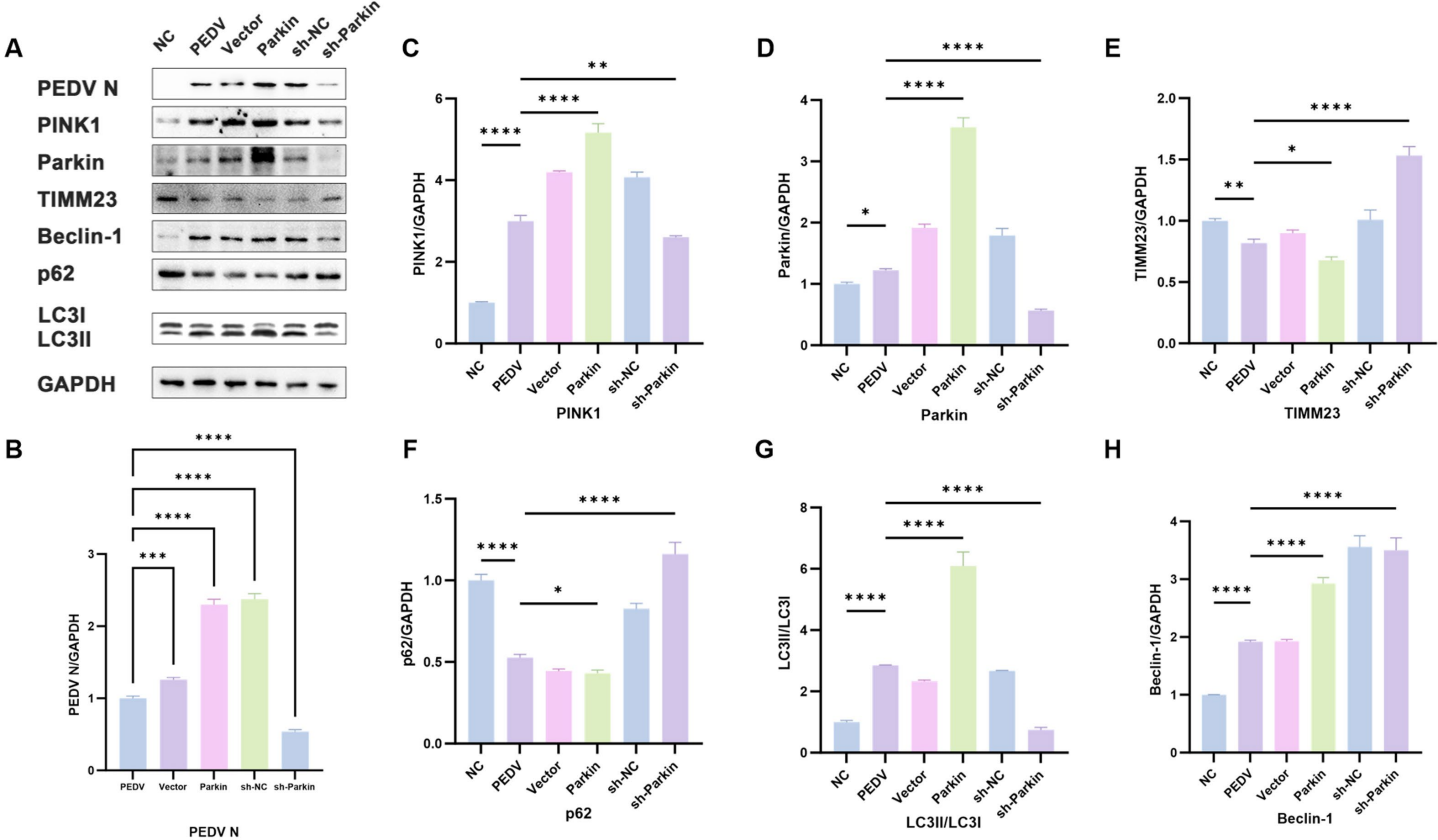
The regulatory role of the PINK1/Parkin pathway in the induction of mitophagy by PEDV. **(A)** Representative Western blot images showing PEDV N, PINK1, Parkin, TIMM23, Be-clin-1, p62, LC3II/I protein levels in Vero cells of each group. **(B)** Relative expression levels of the PEDV N protein in Vero cells in the blank control group, the Parkin overexpression group, and the silencing group, compared to the PEDV-infected group. **(C–H)** The relative expression levels of PINK1, Parkin, TIMM23, p62, LC3II/I, and Beclin-1 in each group of Vero cells. Grayscale values of the protein levels normalized to GAPDH. Each biological replicate was assayed in technical triplicate. Data are presented as mean ± SD. Compared with the control group, were per-formed using a one-way ANOVA analysis. (*p < 0.05, **p < 0.01, ***p < 0.001, ****p < 0.0001).

This study demonstrates that PEDV infection induces PINK1/Parkin-dependent mitophagy. Next, we aim to determine whether the PINK1/Parkin pathway regulates PEDV-induced mitophagy. To achieve this, Parkin was silenced or overexpressed in Vero E6 cells, which were then infected with PEDV at an MOI of 0.1. The expression levels of autophagy-related proteins were assessed by Western blotting (Figure 6A). The results showed that, compared to the control group, Parkin protein expression was significantly increased in the overexpression group and markedly decreased in the siRNA-mediated knockdown group, confirming the successful manipulation of Parkin expression levels in Vero E6 cells (*p* < 0.05; Figure 6B). In addition, overexpression of Parkin would increase the relative expression levels of PINK1 and Parkin proteins, while silencing of Parkin would reduce their relative protein expression levels (*p* < 0.05; Figures 6C,D). Then, Parkin overexpression decreased the protein expression levels of TIMM23 and p62, while Parkin silencing increased the protein expression levels of TIMM23 and p62 (*p* < 0.05; Figures 6E,F). Overexpression of Parkin leads to an increase in the protein expression levels of LC3II/LC3I and Beclin-1, while silencing of Parkin results in a decrease in the LC3II/LC3I ratio and an increase in the relative expression level of Beclin-1 protein (*p* < 0.05; Figures 6G,H). Overall, these results indicate that PEDV-infected cells engage the PINK1/Parkin pathway to regulate PEDV-induced mitophagy.

## 4 Discussion

Mitochondria are a highly efficient biological system for generating energy, which powers eukaryotic cells through biochemical processes. These processes regulate cellular energy homeostasis and cell death (38). Studies have shown that impaired mitochondrial function reduces the organelle’s ability to produce ATP and other substances, leading to elevated and persistent levels of ROS, increased pro-apoptotic factors, and disruption of normal cellular function (39). Therefore, the timely removal of abnormal mitochondria is crucial for cells, and cells have evolved multiple mitochondrial autophagy pathways, including the PINK1/Parkin and Nix/BNIP3 pathways, to ensure that mitochondrial autophagy can be rapidly activated under various conditions (40).

In the context of virus-host interactions, a growing body of experimental evidence suggests that mitophagy plays a significant role in the course of viral infection (41). For instance, studies on the Hepatitis B Virus (HBV), the Hepatitis C Virus (HCV), the Venezuelan Equine Encephalitis Virus (VEEV), the Classical Swine Fever Virus (CSFV), the Porcine Reproductive and Respiratory Syndrome Virus (PRRSV), the Newcastle Disease Virus (NDV), and the Transmissible Gastroenteritis Virus (TGEV) indicate that mitophagy facilitates viral replication and promotes viral infection (33). Regarding PEDV, a member of the Alphacoronavirus genus, existing studies have confirmed that it can cause damage to epithelial cells and induce mitophagy; however, our understanding of the interaction between PEDV infection and autophagy remains limited (36). Therefore, during our study, we selected Vero cells, which are susceptible to PEDV and are commonly used for the isolation and culture of viruses *in vitro*, and confirmed that the mitochondrial structure of infected cells was damaged, autophagosomes appeared, and mitophagy was induced. This indicates that PEDV infection disrupts the host cell’s mitochondria; consequently, we hypothesize that damaged mitochondria undergo autophagy and are subsequently cleared. Since ROS are closely associated with the activation of mitophagy, and studies have shown that ROS generated by mitochondria participate in the induction of autophagy, mitochondrial damage leads to increased ROS production; the excessive accumulation of ROS generated by mitochondria results in the formation of hydrogen peroxide through extracellular superoxide dismutase activity, cause irreversible damage to cells (19, 42, 43). Therefore, we hypothesize that mitochondrial damage and autophagy induce oxidative damage in cells. Experimental results confirm that ROS levels are elevated in PEDV-infected Vero E6 cells, markers of oxidative damage show significant changes, and changes in the expression levels of autophagy-related proteins. Consequently, ROS induce mitophagy and cause oxidative damage in Vero E6 cells, laying the foundation for further studies on the mechanism by which mitophagy affects PEDV replication.

Research on the dynamics of mitochondrial transport indicates that the majority of mitochondrial proteins are encoded by the nucleus and subsequently transported from the cytoplasm to the mitochondria. These proteins are primarily transported through channels formed by complexes of outer membrane transport proteins (TOM) and inner membrane transport proteins (TIM) (44). A decrease in MMP directly impairs the efficiency of transmembrane transport of mitochondrial proteins. This disruption in transport function gradually leads to impaired mitochondrial structural integrity, ultimately resulting in a reduction in the total number of mitochondria within the cell. The present study has determined, through the implementation of experimental results, that during PEDV infection, the MMP of host cells undergoes a significant decrease, while the expression levels of mitochondrial membrane proteins undergo marked changes. These changes in membrane protein expression are closely associated with the activation of mitophagy. Under normal physiological conditions, the balance of mitochondrial numbers within cells is maintained through two dynamic processes: the biosynthesis of new mitochondria and the selective clearance of damaged mitochondria. As is the case with the degradation mechanisms of other damaged organelles or misfolded proteins within the cell, dysfunctional mitochondria are also transported by the cell via the autophagy pathway to lysosomes for degradation (45). The results of this study demonstrate that PEDV infection can directly cause damage to mitochondrial structure and function, leading to the accumulation of ROS within mitochondria and cellular oxidative stress. It is currently understood that the induction of mitophagy involves multiple molecular mechanisms, among which the pathway co-mediated by PINK1 and Parkin plays a central regulatory role in the process of selective mitophagy (46). Research has demonstrated that glycoprotein 5 of PRRSV activates mitochondrial ROS production, thus facilitating viral replication (47). In this study, we obtained analogous results. The accumulation of ROS induced by PEDV-infected cells resulted in the activation of the PINK1/Parkin pathway. Research has demonstrated that a variety of viruses, including HBV, HCV, CSFV, PRRSV, and TGEV, have the capacity to stimulate the process of autophagy within host cells, concurrently impeding the program of apoptosis. This finding stands in contrast to the results of our study. The observed discrepancy in research conclusions may be attributed to differences in the genetic characteristics of PEDV strains and variations in the host cell lines utilized in the experiments. During the dynamic course of viral infection, there is close interaction between viral particles and host cell mitochondria. The results of this study demonstrate that as the duration of PEDV infection increases, the proportion of cells undergoing apoptosis or necrosis exhibits a significant upward trend. Based on this, we hypothesize that in the early stages of infection, PEDV suppresses apoptosis by activating mitophagy, thereby preventing premature host cell death that could interrupt the viral replication cycle; whereas in the late stages of infection, the virus actively induces the apoptosis program to facilitate the release of progeny viral particles from the host cells. Most current research in the academic community has confirmed that virus-induced mitophagy can weaken the host cell’s innate antiviral immune response during infection by regulating signaling pathways within the host cell; this mechanism may be universal across various viral infections. For example, the varicella-zoster virus promotes PINK1/Parkin-mediated mitophagy via glycoprotein E to evade STING- and MAVS-mediated antiviral innate immune responses (48). Our study indicates that mitophagy promotes PEDV replication and is regulated via the PINK1/Parkin pathway. Furthermore, studies have shown that viral replication can be inhibited by suppressing the accumulation of reactive oxygen species, which is consistent with our findings. Viral infection leads to elevated ROS levels, reduced MMP activity, the accumulation of lipid peroxides, and oxidative damage to cells, which in turn triggers mitochondrial damage and subsequent autophagy (49). Mitophagy increases the protein expression levels of PINK1 and Parkin in Vero E6 cells, leading to a significant decrease in the expression levels of mitochondrial inner membrane transporters and adaptor proteins, as well as a significant increase in the LC3II/LC3I ratio and Beclin-1 protein expression. These findings suggest that PEDV regulates mitophagy through the ROS-mediated PINK1/Parkin signaling pathway. Although this study has elucidated some mechanisms by which PEDV induces mitophagy and its relationship with the ROS-mediated classical PINK1/Parkin autophagy pathway, many questions remain unanswered. For example, the specific molecular regulatory mechanisms underlying PEDV’s inhibition of apoptosis in the early stages of infection and its induction of apoptosis in the late stages are still unclear.

### 4.1 Limitations

This study has several limitations. Firstly, all experiments were conducted in Vero E6 cells, which are highly sensitive to PEDV and are not derived from pig sources. Therefore, it is necessary to conduct verification in pig intestinal epithelial cells (such as IPEC-J2 cells) and in *in vivo* models in the future to confirm the universal applicability of the ROS/PINK1/Parkin-mediated mitochondrial autophagy pathway during PEDV infection. Secondly, although our data indicate that ROS accumulation and mitochondrial autophagy activation occur after PEDV infection and are related to enhanced viral replication, this study did not directly distinguish the effects between the viral entry process and the replication events after entry. Further clarification of this issue is needed through methods such as synchronous adsorption–internalization experiments and time addition experiments. Thirdly, we mainly focused on the classical PINK1/Parkin-dependent mitochondrial autophagy pathway because this pathway has been strongly supported in experiments related to mitochondrial membrane potential changes, ROS production, and Parkin function acquisition and loss. Other mitochondrial autophagy pathways, such as the BNIP3/NIX-mediated mechanism, have not been studied. These pathways may also be involved in the interaction between PEDV and the host. Future research should address these issues to more comprehensively understand how PEDV regulates mitochondrial quality control and viral replication.

## 5 Conclusion

In summary, our results indicate that PEDV infection induces structural damage to mitochondria in Vero cells, disrupting redox homeostasis and leading to excessive ROS production and a loss of mitochondrial membrane potential. The PINK1/Parkin pathway senses the loss of membrane potential and mediates ubiquitination of the damaged mitochondria, which subsequently facilitates their recognition and clearance via fusion with autophagosomes. This process creates a favourable environment for PEDV replication and enhances viral propagation efficiency. Therefore, mitochondria-targeted autophagy mediated by the ROS/PINK1/Parkin signal pathway represents a key strategy by which PEDV replicates and exerts cytopathic effects in host cells.

## Data Availability

The datasets presented in this study can be found in online repositories. The names of the repository/repositories and accession number(s) can be found in the article/supplementary material.
